# RIG-1 receptor expression in the pathology of Alzheimer’s disease

**DOI:** 10.1186/1742-2094-11-67

**Published:** 2014-04-02

**Authors:** Juan Pablo de Rivero Vaccari, Frank J Brand, Christina Sedaghat, Deborah C Mash, W Dalton Dietrich, Robert W Keane

**Affiliations:** 1 Department of Neurological Surgery, University of Miami Miller School of Medicine, Miami, FL 33136, USA; 2 Department of Physiology & Biophysics, University of Miami Miller School of Medicine, Miami, FL 33136, USA; 3 Department of Neurology, University of Miami Miller School of Medicine, Miami, FL 33136, USA; 4 Department of Molecular and Cellular Pharmacology, University of Miami Miller School of Medicine, Miami, FL 33136, USA; 5 The Miami Project to Cure Paralysis, University of Miami Miller School of Medicine, Miami, FL 33136, USA

**Keywords:** Innate immunity, Rig signaling, RLR, Inflammation, Alzheimer’s disease, Mild cognitive impairment

## Abstract

**Background:**

Neuroinflammation plays a critical role in the pathogenesis of Alzheimer’s disease (AD) and involves activation of the innate immune response via recognition of diverse stimuli by pattern recognition receptors (PRRs). The inflammatory inducers and precise innate signaling pathway contributing to AD pathology remain largely undefined.

**Results:**

In the present study we analyzed expression levels of innate immune proteins in temporal and occipital cortices from preclinical (no cognitive impairment, NCI, N = 22) to mild cognitive impairment (MCI, N = 20) associated with AD pathology (N = 20) and AD patients (N = 23). We found that retinoic acid-inducible gene-I (RIG-1) is significantly elevated in the temporal cortex and plasma in patients with MCI. In addition, primary human astrocytes stimulated with the RIG-1 ligand 5′ppp RNA showed increased expression of amyloid precursor protein (APP) and amyloid-β (Aβ), supporting the idea that RIG-1 is involved in the pathology of MCI associated with early progression to AD.

**Conclusion:**

These findings suggest that RIG-1 may play a critical role in incipient AD.

## Background

Alzheimer’s disease (AD) pathogenesis is associated with central nervous system (CNS) inflammatory responses [1-4]. Amyloid-β (Aβ) fibrils trigger inflammatory responses mediated by Toll-like receptors (TLR)4/TLR6 in the presence of CD36 [1-4]. Moreover, a polymorphism in the TLR4 extracellular domain has been reported to be associated with protection against late-onset AD in an Italian population [5], suggesting that a sterile inflammatory response could influence AD pathology through TLR4 signaling. In addition, TLR2 has been shown to act as a receptor for Aβ, and to trigger an inflammatory response [6]. Activation of innate immunity in the CNS appears to be a universal component of neuroinflammation. AD may be distinguished by a disease-specific mechanism for induction of inflammatory responses. In addition, distinct pathways for production of inflammation inducers in vulnerable brain regions where these processes occur are potential biomarkers of AD pathophysiology.

Infection of cells by viruses and microorganisms activates innate immune inflammatory responses. The initial sensing of infection is mediated by pattern recognition receptors, which include TLRs, RIG-I-like receptors (RLR), NOD-like receptors (NLR), and C-type lectin receptors (CLR). The RLR family is a RNA sensing system that is comprised of retinoic acid inducible gene-like-I (RIG-1), melanoma differentiation-associated gene 5 (MDA5), and laboratory of genetics and physiology 2 (LGP2). RIG-1 recognizes relatively short dsRNA (up to 1 kb) whereas MDA5 detects long dsRNA (more than 2 kb) to activate synthesis of type I IFNs, including IFN-α and IFN-β [7]. RLRs are localized in the cytoplasm and recognize the genomic RNA of dsRNA viruses and dsRNA generated as the replication intermediate of ssRNA viruses and also act as sensors of cellular damage [8]. RLRs activate downstream signaling proteins evoking type I IFN production. Type I IFNs play central roles in antiviral responses by inducing apoptotic cell death in virally infected cells, rendering cells resistant to virus infection, activating acquired immunity, and stimulating hematopoietic stem cell turnover and proliferation. In addition, type I IFNs have been implicated in the inflammatory response in AD [9].

We have shown recently that RLR signaling proteins are present in CNS neurons and glial cells, and RLR signaling stimulation resulted in astrocyte activation [10]. In addition, activation of the inflammasome, an NLR innate immune complex, contributes to age-related cognitive decline in elderly animals [11]. However, limited information is available about the role of RLRs in AD pathology or early disease progression. Since MCI is considered a transitional phase between normal aging (or cognition) and AD [12-14], it is important to identify the molecular events that characterize MCI associated with AD pathology.

## Methods

### Patient consents and subjects demographics

The study was approved by the University of Miami Miller School of Medicine institutional review board. Written informed consent for research and brain autopsy was obtained for all subjects in this study.

Neuropathologic specimens (3 millimeters) of fresh-frozen human temporal (BA38) and occipital cortex (BA17) were obtained from the University of Miami Brain Endowment Bank™. The temporopolar cortex (BA38) was sampled from frozen tissue blocks at the level of the fundus of the temporopolar sulcus. The occipital cortex was sampled from the primary visual cortex (BA17). Postmortem specimens were selected from age-matched subjects with no cognitive impairment (NCI), MCI, and from AD patients. The diagnosis of AD was made using standard diagnostic criteria [15]. Subjects with NCI, MCI, and AD were selected based on their antemortem clinical dementia rating (CDR) score one year prior to death and postmortem pathologic evaluation for AD pathology and Braak stage. Neuropathologic diagnosis was based on NIA-Regan criteria recommendations of the Consortium to Establish a Registry for AD (CERAD) [16] and Braak staging of neurofibrillary tangles [17]. The diagnosis of MCI included assessment of normal activities of daily living, normal general cognitive function, abnormal memory for age, and no dementia [17]. MCI patients met neuropathologic criteria for possible to probable AD and Braak stages I to IV [17]. AD cases selected for this study included patients with a diagnosis of clinical dementia and definite AD on postmortem examination (Braak stages V or VI; Table 1).

**Table 1:** Characteristics of subjects used in the study (brain cortex)

**Characteristic**	**NCI**	**MCI**	**AD**
Number of subjects	22	20	23
Male (%)	15 (67)	4 (19)	9 (36)
Female (%)	7 (33)	16 (81)	14 (64)
Age at death			
Median (IQR)	68 (61 to 79)	86 (70 to 91)	80 (70 to 85)
Range	59 to 95	61 to 105	60 to 88
Race	20C 1H	20C 1H	24C 1H
Brain weight			
Median (IQR)	1,352 (1,298 to 1,505)	1,210 (1,043 to 1,398)	1,115 (950 to 1,215)
Range	1,054 to 1,570	880 to 1,840	825 to 1,250
CDR score			
0 (%)	22 (100)	12 (60)	0 (0)
1 (%)	0 (0)	8 (40)	0 (0)
2 (%)	0 (0)	0 (0)	2 (9)
3 (%)	0 (0)	0 (0)	21 (91)
Braak score			
0 (%)	22 (100)	0 (0)	0 (0)
I (%)	0 (0)	6 (30)	0 (0)
II (%)	0 (0)	5 (25)	0 (0)
III (%)	0 (0)	9 (45)	0 (0)
IV (%)	0 (0)	0 (0)	1 (4)
V (%)	0 (0)	0 (0)	13 (56)
VI (%)	0 (0)	0 (0)	9 (40)
AD CERAD			
Not present (%)	22 (100)	1 (5)	0 (0)
Possible (%)	0 (0)	4 (20)	0 (0)
Probable (%)	0 (0)	5 (25)	0 (0)
Definite (%)	0 (0)	10 (50)	23 (100)

*AD*: Alzheimer’s disease, *CDR*: clinical dementia rating, *CERAD*: Consortium to Establish a Registry for Alzheimer’s Disease, *IQR*: interquartile range, *MCI*: mild cognitive impairment, *NCI*: no cognitive impairment. Race: *C* = Caucasian and *H* = Hispanic.

### Plasma and serum samples

All plasma and serum samples were obtained from the University of Kentucky Alzheimer’s Disease Center Brain Bank. The samples were obtained from patients diagnosed postmortem as either age-matched controls with no cognitive impairment (NCI; Braak stage (0 to I), MCI (Braak stages II to IV), or AD (Braak stages V to VI). The section of the study included six age-matched controls (NCI; Braak stages 0 to I), seven MCI patients with possible AD, determined by pathological evidence of neurofibrillary tangles and senile plaques (Braak stages II to IV), and ten patients who met clinical diagnostic criteria for definite AD (Braak stages V to VI; Table 2).

**Table 2:** Characteristics of subjects used in the study (plasma and serum)

**Group**	**Braak stage**	**AD (CERAD)**	**Age at death**	**Gender**	***Apoe***	**PMI (hours)**
NCI	0	B = CERAD Probable	92	M	3/5	3.33
NCI	0	No	85	F	3/3	2.50
NCI	1	No	90	F	2/3	4.00
NCI	1	No	100	F	2/3	2.25
NCI	1	No	84	F	3/4	3.00
NCI	1	No	79	F	3/4	1.75
MCI	2	B = CERAD Probable	91	F	3/4	1.75
MCI	2	B = CERAD Probable	93	F	3/4	2.75
MCI	2	B = CERAD Probable	80	F	3/4	2.00
MCI	2	A = CERAD Probable	81	M	3/5	2.83
MCI	2	C = Definite AD	79	M	3/3	1.75
MCI	4	B = CERAD Probable	77	M	3/4	2.75
MCI	3	B = CERAD Probable	92	F	2/3	3.25
AD	6	C = Definite AD	78	M	3/4	3.50
AD	6	C = Definite AD	84	M	3/4	2.75
AD	6	C = Definite AD	83	F	3/3	3.50
AD	6	B = CERAD Probable	85	M	3/3	2.75
AD	6	B = CERAD Probable	80	M	3/3	2.75
AD	6	C = Definite AD	87	M	3/4	3.25
AD	6	C = Definite AD	73	M	3/3	2.00
AD	6	C = Definite AD	80	F	3/3	4.00
AD	6	C = Definite AD	83	F	3/4	2.25
AD	5	C = Definite AD	91	F	3/3	3.00

*AD*: Alzheimer’s disease, *Apoe*, apolipoprotein e; *CERAD*: Consortium to Establish a Registry for AD: *MCI*: mild cognitive impairment, *NCI*: no cognitive impairment, *PMI*: postmortem interval.

### Plasma and serum immunoglobulin isolation

To prevent interference of immunoglobulin G (IgG) during immunoblot analysis of plasma and serum, IgG was isolated using a Pierce Albumin/IgG Removal kit (Thermo Scientific Waltham, MA, USA) according to manufacturer’s instructions.

### Immunoblotting

**Figure 1:**
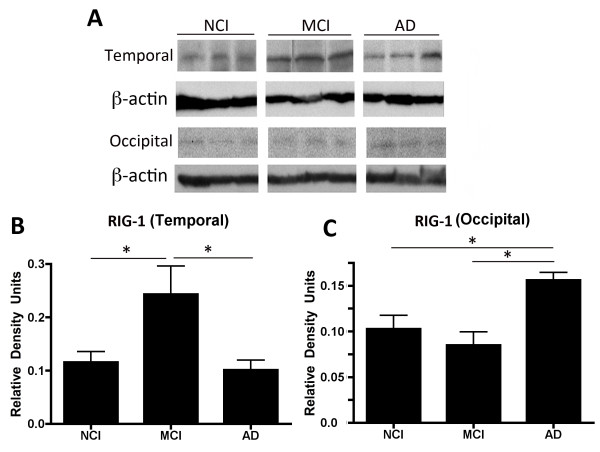
**RIG-1 is elevated in the temporal cortex of mild cognitive impairment (MCI) patients.** Representative immunoblots **(A)** of the temporal cortex **(B)** and occipital cortex **(C)** from age-matched controls (NCI), MCI and Alzheimer Disease (AD) patients analyzed for RIG-1 expression. β-actin was used as a protein loading control and internal standard. Data are presented as mean ± SEM. *P < 0.05. N = NCI: 22, MCI: 20 and AD: 23.

**Figure 2:**
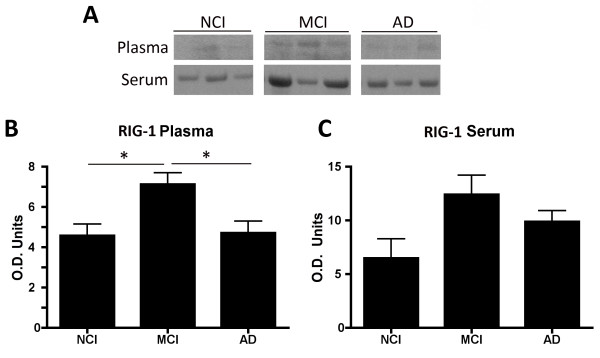
**RIG-1 is elevated in the plasma of mild cognitive impairment (MCI) patients.** Representative immunoblots **(A)** of plasma **(B)** and serum **(C)** from age-matched controls (NCI), MCI and Alzheimer Disease (AD) patients analyzed for RIG-1 expression. 5 μg of protein were loaded for the plasma and serum samples after removal of IgG. Data presented as mean ± SEM. *P < 0.05. N = NCI: 6, MCI: 7 and AD: 10 patients.

Occipital and temporal cortices were homogenized in lysis buffer (20 mM Tris, pH 7.5, 150 mM NaCl, 1 mM EDTA, 1 mM EGTA, 1% Triton X-100, 2.5 mM pyrophosphate, 1 mM β-glycerophosphate) with protease inhibitor cocktail (Sigma). Twenty five micrograms of protein per sample were resolved in 10 to 20% Tris-HCl Criterion precasted gels (Bio-Rad, Hercules, CA, USA), transferred to polyvinylidene difluoride membranes (Applied Biosystems Waltham, MA, USA) and placed in blocking buffer (PBS, 0.1% Tween-20, 0.4% I-Block (Applied Biosystems Waltham, MA, USA) and then incubated for one hour with an antibody against RIG-1 (Anaspec Fremont, CA, USA) at a dilution of 1:1,000. To authenticate the presumptive bands shown in Figures 1 and 2, a RIG-1 positive control sample (Novus Biologicals Littleton, CO, USA) was used. Immunoabsorption is more appropriate to demonstrate the authenticity of the bands. Membranes were incubated for one hour with primary antibodies followed by appropriate secondary horseradish peroxidase (HRP)-linked antibodies (Cell Signaling Danvers, MA, USA). Visualization of signal was enhanced by chemiluminescence using a Phototope-HRP detection kit (Cell Signaling Danvers, MA, USA). To control for protein loading, immunoblots were stripped with Restore, Western blot stripping buffer (Pierce Rockford, IL, USA) and blotted for β-actin using monoclonal anti-β-actin antibody (1:8,000, Sigma St. Louis, MO, USA). Quantification of band density was performed using the UN-SCAN-IT gel software, and data were normalized to β-actin. For immunoblotting of serum and plasma 5 μg of protein were loaded equally across all samples used to keep data normalized.

### Astrocyte culture preparation and RIG-1 stimulation

Human astrocytes were grown in culture as described in de Rivero Vaccari *et al*. in 2012 [10]. Primary human astrocytes (Lonza Basel, Switzerland) were grown in culture in complete Astrocyte Growth Medium (Lonza Basel, Switzerland) for seven days. RIG-1 signaling was stimulated with 5′ triphosphate double-stranded RNA (5′ppp dsRNA, Invivogen San Diego, CA, USA) as a specific ligand to stimulate RIG-1 signaling at different concentrations (2 and 4 μg/ml) for 18 hours. After stimulation, cells were harvested and immunoblotted for RIG-1 (Anaspec Fremont, CA, USA), phosphorylated IRF3 (Novus Biologicals Littleton, CO, USA), amyloid precursor protein (Abcam Cambridge, MA, USA) and amyloid-β (Epitomics Burlingame, CA, USA) expression as described.

### Stimulation of human astrocytes with 3-42 amyloid-β

Human astrocytes were grown in culture for seven days and stimulated with 3-42 amyloid-β (Anaspec Fremont, CA USA) at a concentration of 0.5, 1 and 3 μM for 18 hours. Then cells were harvested and immunoblotted for expression of caspase-1 (Imgenex San Diego, CA, USA) and RIG-1 (Anaspec Fremont, CA USA) as described.

### Statistical analysis

The primary outcome measures were levels of immune proteins in two brain regions. The demographic, clinical and neuropathological characteristics were used to group assignment. Association between individual protein measures and age, gender or postmortem interval were explored in multivariate analyses to ensure that the results were unchanged. Statistical comparisons between groups were made using one-way ANOVA and one-tailed Student’s *t*-test. The level of statistical significance was set at * *P* < 0.05.

## Results

### RIG-1 is elevated in the temporal cortex of MCI patients

The demographic and neuropathology characteristics of the cohort used in this section of the study are summarized in Table 1. The study included 22 age-matched controls (NCI), 20 MCI patients with pathologic evidence of senile plaques and neurofibrillary tangles consistent with possible or probable AD (Braak stages I to IV), and 23 patients who met clinical diagnostic criteria for AD and definite pathologic evidence (Braak V to VI). Immunoblot analysis of temporal cortical samples revealed an increase in RIG-1 expression in the MCI group when compared to the NCI and AD groups (Figure 1B). In contrast, the levels of RIG-1 in the occipital cortex were higher in the AD group than in the NCI and MCI groups (Figure 1C). Thus, these results show for the first time that RIG-1 is increased in the temporopolar cortex of MCI patients.

### RIG-1 is elevated in the plasma of MCI patients

To determine the levels of RIG-1 in the plasma and serum of patients with MCI associated with AD, immunoglobulin G was isolated from serum and plasma obtained from patients corresponding to the NCI, MCI and AD groups, as described above. Figure 2 shows that RIG-1 was significantly increased in the plasma (Figure 2B) from MCI patients compared to the NCI and AD groups, whereas the levels of RIG-1 in serum (Figure 2C) did not differ among the three groups. Thus, these results show for the first time that RIG-1 is increased in the plasma of MCI patients.

### 3-42 Aβ increases expression of RIG-1

**Figure 3:**
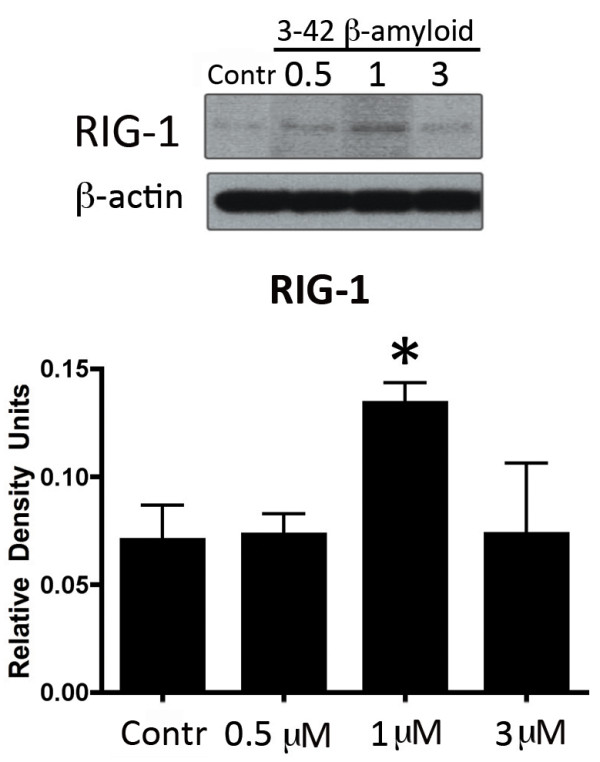
**3-42 Aβ increases expression of RIG-1.** Representative immunoblot analysis of human cortical astrocyte lysates of cells stimulated with 0.5, 1 and 3 μM of 3-42 Aβ for 18 hours. Non-stimulated cells were used as a control (Contr). Cell lysates were immunoblotted with antibodies against RIG-1. β-Actin was used as internal standard and control for protein loading. Data presented as mean ± SEM. *P < 0.05. N = 6.

3-42 Aβ species have been shown to be the most prevalent form of Aβ peptides present in early and later stages of human AD amyloid pathology [18]. Since we found that levels of RIG-1 expression are elevated in the temporal cortex from MCI patients when compared to end-stage AD pathology (AD, Figures 1 and 2), we stimulated human cortical astrocytes with 3-42 Aβ for 18 hours at different concentrations (C, 0.5, 1 and 3 μM) to determine if Aβ peptide levels regulate the protein expression levels of RIG-1. Interestingly, there was a concentration dependent effect of 3-42 Aβ on the expression of RIG-1. At 0.5 μM treatment, the RIG-1 levels did not change when compared to the control/untreated group, whereas at 1 μM, the levels of RIG-1 increased, and at 3 μM, the protein levels of RIG-1 returned to basal/control levels (Figure 3). Importantly, no morphological or toxic changes were noticed in the cultured astrocytes at the concentrations of 3-42 Aβ used for 18 hours (data not shown). Thus, it appears that Aβ may be involved in regulating the levels of the RIG-1 protein.

### 5′ppp dsRNA activates RIG-1 signaling in primary human cortical astrocytes

**Figure 4:**
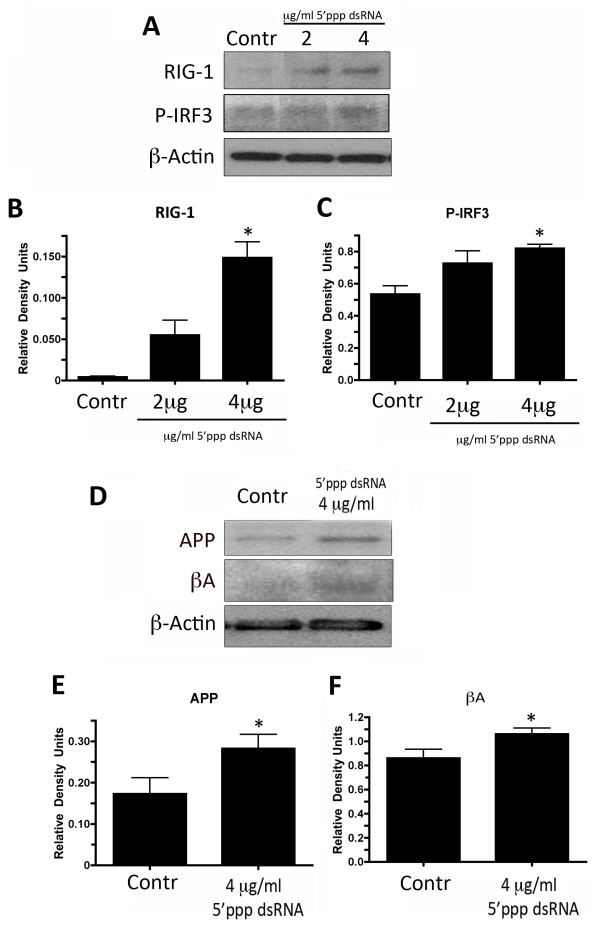
**5′ppp dsRNA activates RIG-1 signaling and increases expression of APP and Aβ.** Representative immunoblot analysis of human cortical astrocyte lysates **(A)** of cells stimulated with 2 or 4 μg/ml of 5′ppp dsRNA for 18 hours. Non-stimulated cells were used as a control (Contr). Cell lysates were immunoblotted with antibodies against **(B)** RIG-1 and **(C)** P-IRF3. β-Actin was used as internal standard and control for protein loading. Data presented as mean ± SEM. *P < 0.05. N = 6. Representative immunoblot analysis of human cortical astrocyte lysates **(D)** of cells stimulated with 4 μg/ml of 5′ppp dsRNA for 18 hours. Non-stimulated cells were used as a control (Contr). Cell lysates were immunoblotted with antibodies against **(E)** APP and **(F)** Aβ. β-Actin was used as internal standard and control for protein loading. Data presented as mean ± SEM. *P < 0.05. N = 6.

5′ppp dsRNA has been shown to be a specific ligand of RIG-1 signaling activation [19]. To determine whether 5′ppp dsRNA is responsible for the activation of RIG-1 in primary human cortical astrocytes, 5′ppp dsRNA was administered to primary astrocytes in culture for 18 hours at two different concentrations (2 and 4 μg/ml). As shown in Figure 4B and 4C, RIG-1 and phospho-interferon regulatory factor 3 (P-IRF3), respectively, were significantly elevated after the administration of 4 μg/ml of 5′ppp dsRNA, thus indicating RIG-1 signaling activation.

### 5′ppp dsRNA increases expression of APP and Aβ in primary human cortical astrocytes

To identify if RIG-1 signaling stimulation is involved in the pathogenesis of AD, astrocytes were stimulated with the RIG-1 signaling agonist 5′ppp dsRNA (4 μg/ml) for 18 hours. Samples were then resolved by immunoblotting using antibodies against two hallmark proteins of AD, APP (Figure 4E) and Aβ (Figure 4F). Stimulation of RIG-1 with 4 μg/ml 5′ppp dsRNA, which activates RIG-1 signaling in astrocytes, resulted in a significant elevation in the expression of APP and Aβ when compared to the control group, suggesting an involvement of RIG-1 signaling in the expression of two hallmark proteins in AD pathology.

## Discussion

The results of the present study demonstrate that RIG-1 is significantly elevated in the plasma and temporal cortex of MCI patients with AD pathology whereas RIG-1 is elevated in the occipital cortex of AD patients. Stimulation of RIG-1 with 5′ppp dsRNA in human cortical astrocytes resulted in increased expression of APP and Aβ. Thus, these findings suggest a potential role of the RIG-1 signaling system in incipient AD.

AD is a progressive neurodegenerative disorder characterized by impaired judgment, confusion, changes in behavior, disorientation [20], impairment of daily living, and loss of the ability to function independently [21]. AD is expected to become more prevalent as life expectancy continues to rise. It has been estimated that by 2050, the number of AD cases could double or triple to between 11 to 16 million [22]. A major limitation in finding therapeutic solutions for AD has been the lack of reliable methods for early diagnosis of this devastating disease. AD is a neurodegenerative disorder characterized by a progressive cognitive impairment as a consequence of neuronal dysfunction and ultimately the death of neurons. MCI is considered a transitional phase between normal aging and AD [12-14]. The amyloid hypothesis of AD proposes that neuronal damage results from the accumulation of insoluble, hydrophobic, fibrillar peptides such as amyloid-β_1-42_[23-26]. These peptides activate enzymes resulting in a cascade of second messengers including prostaglandins and platelet-activating factor. Apoptosis of neurons is thought to follow as a consequence of the uncontrolled release of second messengers. It is possible that RIG-1 signaling in the temporal cortex is involved in the early events leading to AD pathology such as the accumulation of APP. On the other hand, the presence of RIG-1 in the occipital cortex of AD patients may be associated with exacerbated production of cytokines in AD patients [27] as a result of disease progression in later stages of AD when the pathology spreads throughout the cortex from the limbic to koniocortical areas.

Neuroinflammation has been considered to play a critical role in the pathogenesis of AD [28-33], but the role of the innate immune response has not been thoroughly examined [34,35]. Human neurons, in the absence of glia, have the intrinsic machinery to trigger robust inflammatory, chemoattractive, and antiviral responses [36]. The innate immune system senses microbial and viral pathogen and danger signals released from damaged or stressed cells to trigger conserved intracellular signaling pathways that drive proinflammatory responses that are critical for productive innate and adaptive immunity. Excessive inflammatory responses become deleterious adding to tissue destruction. Here we have provided evidence demonstrating that the RIG-1 is elevated in the innate immune response in disease-affected brain areas of MCI patients.

RIG-1 signaling may be activated by small self-RNA cleavage products generated by RNase L that stimulate signaling of RIG-1 [37] or by reactive oxygen species (ROS) [38]. Since damaged CNS cells release small self-nucleic acids and ROS, these molecules may play an important role in the initiation of the innate immune response in MCI [39]. Alternatively, foreign nucleic acids, the signature of invading viruses and certain bacteria, are sensed intracellularly and then stimulate RIG-1 signaling [7]. Other, yet to be identified ligands may be involved in the activation of RIG-1 signaling in MCI. Moreover, our data suggest that RIG-1 signaling activation results in increased expression of APP and Aβ, and that in addition Aβ contributes to the expression of RIG-1. It is important to consider that this study used samples from individuals in the MCI group that had a slightly greater number of females and a wider age range; thus, when interpreting these results one must take into account the effects of gender and age [40].

## Conclusions

In this study, we used immunoblot analysis to determine whether RIG-1 signaling stimulation results in increased expression of Aβ and APP. In order to determine whether human cortical astrocytes respond to RIG-1 stimulation, we treated primary cortical astrocytes in culture with the specific RIG-1 ligand 5′ppp dsRNA and assayed for the expression of the RIG-1 signaling proteins RIG-1 and P-IRF3.

as well as APP and Aβ. The levels of these proteins were increased upon stimulation with the RIG-1 ligand, consistent with the hypothesis that RIG-1 signaling is involved in the pathogenesis of AD. Astrocytes have been previously implicated in the pathogenesis of AD [41-44]. In addition, we have previously shown that RIG-1 signaling is involved in the activation of astrocytes [10]. Thus, our findings further support an involvement of astrocytes in AD pathology.

## Abbreviations

AD: Alzheimer’s disease; PRRs: pattern recognition receptors; NCI: no cognitive impairment; MCI: mild cognitive impairment; RIG-1: retinoic acid-inducible gene-I; APP: amyloid precursor protein; Aβ: amyloid-β; CNS: central nervous system; RLR: RIG-I-like receptors; NLR: NOD-like receptors; CLR: C-type lectin receptors; MDA5: melanoma differentiation-associated gene 5; LGP2: laboratory of genetics and physiology 2; CDR: clinical dementia rating; CERAD: Consortium to Establish a Registry for AD; Apoe: apolipoprotein e; 5′ppp dsRNA: 5′ triphosphate double-stranded RNA.

## Competing interests

The authors declare that they have no competing interests.

## Authors’ contributions

JPdRV designed the study, performed experiments, analyzed the data, interpreted the results and prepared the manuscript. FJB and CS performed experiments and analyzed the data. DCM, WDD and RWK reviewed and discussed the manuscript. All authors have read and approved the final version of the manuscript.

## References

[B1] Vollmar P Kullmann JS Thilo B Claussen MC Rothhammer V Jacobi H Sellner J Nessler S Korn T Hemmer B Active immunization with amyloid-beta 1-42 impairs memory performance through TLR2/4-dependent activation of the innate immune system J Immunol 2010 185 6338 6347 10.4049/jimmunol.1001765 20943998

[B2] Stewart CR Stuart LM Wilkinson K van Gils JM Deng J Halle A Rayner KJ Boyer L Zhong R Frazier WA Lacy-Hulbert A El Khoury J Golenbock DT Moore KJ CD36 ligands promote sterile inflammation through assembly of a toll-like receptor 4 and 6 heterodimer Nat Immunol 2010 11 155 161 10.1038/ni.1836 20037584 PMC2809046

[B3] Tang SC Lathia JD Selvaraj PK Jo DG Mughal MR Cheng A Siler DA Markesbery WR Arumugam TV Mattson MP Toll-like receptor-4 mediates neuronal apoptosis induced by amyloid beta-peptide and the membrane lipid peroxidation product 4-hydroxynonenal Exp Neurol 2008 213 114 121 10.1016/j.expneurol.2008.05.014 18586243 PMC2597513

[B4] Jin JJ Kim HD Maxwell JA Li L Fukuchi K Toll-like receptor 4-dependent upregulation of cytokines in a transgenic mouse model of Alzheimer’s disease J Neuroinflammation 2008 5 23 10.1186/1742-2094-5-23 18510752 PMC2430555

[B5] Minoretti P Politi P Coen E Di Vito C Bertona M Bianchi M Emanuele E The T393C polymorphism of the GNAS1 gene is associated with deficit schizophrenia in an Italian population sample Neurosci Lett 2006 397 159 163 10.1016/j.neulet.2005.12.028 16406317

[B6] Liu S Liu Y Hao W Wolf L Kiliaan AJ Penke B Rube CE Walter J Heneka MT Hartmann T Menger MD Fassbender K TLR2 is a primary receptor for Alzheimer’s amyloid beta peptide to trigger neuroinflammatory activation J Immunol 2012 188 1098 1107 10.4049/jimmunol.1101121 22198949

[B7] Wilkins C Gale M Jr Recognition of viruses by cytoplasmic sensors Curr Opin Immunol 2010 22 41 47 10.1016/j.coi.2009.12.003 20061127 PMC3172156

[B8] Takeuchi O Akira S Pattern recognition receptors and inflammation Cell 2010 140 805 820 10.1016/j.cell.2010.01.022 20303872

[B9] Taylor JM Minter MR Newman AG Zhang M Adlard PA Crack PJ Type-1 interferon signaling mediates neuro-inflammatory events in models of Alzheimer’s disease Neurobiol Aging 2014 35 1012 1023 10.1016/j.neurobiolaging.2013.10.089 24262201

[B10] de Rivero Vaccari JP Minkiewicz J Wang X De Rivero Vaccari JC German R Marcillo AE Dietrich WD Keane RW Astrogliosis involves activation of retinoic acid-inducible gene-like signaling in the innate immune response after spinal cord injury Glia 2012 60 414 421 10.1002/glia.22275 22161971 PMC3265608

[B11] Mawhinney LJ de Vaccari Rivero JP Dale GA Keane RW Bramlett HM Heightened inflammasome activation is linked to age-related cognitive impairment in fischer 344 rats BMC Neurosci 2011 12 123 10.1186/1471-2202-12-123 22133203 PMC3259063

[B12] Morris JC Storandt M Miller JP McKeel DW Price JL Rubin EH Berg L Mild cognitive impairment represents early-stage Alzheimer disease Arch Neurol 2001 58 397 405 11255443 10.1001/archneur.58.3.397

[B13] Mufson EJ Binder L Counts SE DeKosky ST de Toledo-Morrell L Ginsberg SD Ikonomovic MD Perez SE Scheff SW Mild cognitive impairment: pathology and mechanisms Acta Neuropathol 2012 123 13 30 10.1007/s00401-011-0884-1 22101321 PMC3282485

[B14] Petersen RC Smith GE Waring SC Ivnik RJ Tangalos EG Kokmen E Mild cognitive impairment: clinical characterization and outcome Arch Neurol 1999 56 303 308 10.1001/archneur.56.3.303 10190820

[B15] Fleming KC Adams AC Petersen RC Dementia: diagnosis and evaluation Mayo Clin Proc 1995 70 1093 1107 10.4065/70.11.1093 7475341

[B16] Mirra SS Heyman A McKeel D Sumi SM Crain BJ Brownlee LM Vogel FS Hughes JP van Belle G Berg L The consortium to establish a registry for Alzheimer’s disease (CERAD): part II standardization of the neuropathologic assessment of Alzheimer’s disease Neurology 1991 41 479 486 10.1212/WNL.41.4.479 2011243

[B17] Braak H Braak E Neuropathological staging of Alzheimer-related changes Acta Neuropathol 1991 82 239 259 10.1007/BF00308809 1759558

[B18] Schieb H Kratzin H Jahn O Mobius W Rabe S Staufenbiel M Wiltfang J Klafki HW Beta-amyloid peptide variants in brains and cerebrospinal fluid from amyloid precursor protein (APP) transgenic mice: comparison with human Alzheimer amyloid J Biol Chem 2011 286 33747 33758 10.1074/jbc.M111.246561 21795681 PMC3190810

[B19] Hornung V Ellegast J Kim S Brzozka K Jung A Kato H Poeck H Akira S Conzelmann KK Schlee M Endres S Hartmann G 5′-triphosphate RNA is the ligand for RIG-I Science 2006 314 994 997 10.1126/science.1132505 17038590

[B20] Thies W Bleiler L Alzheimer’s disease facts and figures Alzheimers Dement 2011 2011 7 208 244 21414557 10.1016/j.jalz.2011.02.004

[B21] Salloway S Mintzer J Weiner MF Cummings JL Disease-modifying therapies in Alzheimer’s disease Alzheimers Dement 2008 4 65 79 10.1016/j.jalz.2007.10.001 18631951

[B22] Hebert LE Scherr PA Bienias JL Bennett DA Evans DA Alzheimer disease in the US population: prevalence estimates using the 2000 census Arch Neurol 2003 60 1119 1122 10.1001/archneur.60.8.1119 12925369

[B23] Lorenzo A Yuan M Zhang Z Paganetti PA Sturchler-Pierrat C Staufenbiel M Mautino J Vigo FS Sommer B Yankner BA Amyloid beta interacts with the amyloid precursor protein: a potential toxic mechanism in Alzheimer’s disease Nat Neurosci 2000 3 460 464 10.1038/74833 10769385

[B24] Yankner BA New clues to Alzheimer’s disease: unraveling the roles of amyloid and tau Nat Med 1996 2 850 852 10.1038/nm0896-850 8705846

[B25] Yankner BA The pathogenesis of Alzheimer’s disease: is amyloid beta-protein the beginning or the end? Ann N Y Acad Sci 2000 924 26 28 11193797 10.1111/j.1749-6632.2000.tb05555.x

[B26] Yankner BA Lu T Amyloid beta-protein toxicity and the pathogenesis of Alzheimer disease J Biol Chem 2009 284 4755 4759 10.1074/jbc.R800018200 18957434 PMC2643502

[B27] Ricci S Fuso A Ippoliti F Businaro R Stress-induced cytokines and neuronal dysfunction in Alzheimer’s disease J Alzheimers Dis 2012 28 11 24 22124029 10.3233/JAD-2011-110821

[B28] Rogers J Inflammation as a pathogenic mechanism in Alzheimer’s disease Arzneimittelforschung 1995 45 439 442 7763341

[B29] Rogers J The inflammatory response in Alzheimer’s disease J Periodontol 2008 79 1535 1543 10.1902/jop.2008.080171 18673008

[B30] Rogers J Shen Y A perspective on inflammation in Alzheimer’s disease Ann N Y Acad Sci 2000 924 132 135 11193789 10.1111/j.1749-6632.2000.tb05571.x

[B31] Rogers JT Leiter LM McPhee J Cahill CM Zhan SS Potter H Nilsson LN Translation of the Alzheimer amyloid precursor protein mRNA is up-regulated by interleukin-1 through 5′-untranslated region sequences J Biol Chem 1999 274 6421 6431 10.1074/jbc.274.10.6421 10037734

[B32] Simard AR Rivest S Neuroprotective properties of the innate immune system and bone marrow stem cells in Alzheimer’s disease Mol Psychiatry 2006 11 327 335 10.1038/sj.mp.4001809 16491130

[B33] Weninger SC Yankner BA Inflammation and Alzheimer disease: the good, the bad, and the ugly Nat Med 2001 7 527 528 10.1038/87839 11329045

[B34] McGeer PL Rogers J McGeer EG Inflammation, anti-inflammatory agents and Alzheimer disease: the last 12 years J Alzheimers Dis 2006 9 271 276 16914866 10.3233/jad-2006-9s330

[B35] Shen Y Lue L Yang L Roher A Kuo Y Strohmeyer R Goux WJ Lee V Johnson GV Webster SD Cooper NR Bradt B Rogers J Complement activation by neurofibrillary tangles in Alzheimer’s disease Neurosci Lett 2001 305 165 168 10.1016/S0304-3940(01)01842-0 11403931

[B36] Lafon M Megret F Lafage M Prehaud C The innate immune facet of brain: human neurons express TLR-3 and sense viral dsRNA J Mol Neurosci 2006 29 185 194 10.1385/JMN:29:3:185 17085778

[B37] Malathi K Paranjape JM Bulanova E Shim M Guenther-Johnson JM Faber PW Eling TE Williams BR Silverman RH A transcriptional signaling pathway in the IFN system mediated by 2′-5′-oligoadenylate activation of RNase L Proc Natl Acad Sci USA 2005 102 14533 14538 10.1073/pnas.0507551102 16203993 PMC1239948

[B38] Tal MC Sasai M Lee HK Yordy B Shadel GS Iwasaki A Absence of autophagy results in reactive oxygen species-dependent amplification of RLR signaling Proc Natl Acad Sci USA 2009 106 2770 2775 10.1073/pnas.0807694106 19196953 PMC2650341

[B39] Rainer TH Lam NY Circulating nucleic acids and critical illness Ann N Y Acad Sci 2006 1075 271 277 10.1196/annals.1368.035 17108220

[B40] Kitamura T Kitamura M Hino S Tanaka N Kurata K Gender differences in clinical manifestations and outcomes among hospitalized patients with behavioral and psychological symptoms of dementia J Clin Psychiatry 2012 73 1548 1554 10.4088/JCP.11m07614 23290328

[B41] Allaman I Gavillet M Belanger M Laroche T Viertl D Lashuel HA Magistretti PJ Amyloid-beta aggregates cause alterations of astrocytic metabolic phenotype: impact on neuronal viability J Neurosci 2010 30 3326 3338 10.1523/JNEUROSCI.5098-09.2010 20203192 PMC6634099

[B42] Calvillo M Diaz A Limon DI Mayoral MA Chanez-Cardenas ME Zenteno E Montano LF Guevara J Espinosa B Amyloid-beta induces a permanent phosphorylation of HSF-1, but a transitory and inflammation-independent overexpression of Hsp-70 in C6 astrocytoma cells Neuropeptides 2013 47 339 346 10.1016/j.npep.2013.06.002 23850171

[B43] Grolla AA Sim JA Lim D Rodriguez JJ Genazzani AA Verkhratsky A Amyloid-beta and Alzheimer’s disease type pathology differentially affects the calcium signalling toolkit in astrocytes from different brain regions Cell Death Dis 2013 4 e623 10.1038/cddis.2013.145 23661001 PMC3674354

[B44] Nagele RG D’Andrea MR Lee H Venkataraman V Wang HY Astrocytes accumulate a beta 42 and give rise to astrocytic amyloid plaques in Alzheimer disease brains Brain Res 2003 971 197 209 10.1016/S0006-8993(03)02361-8 12706236

